# Supplementary figures and images for: Growth on stiffer substrates impacts animal health and longevity in C. elegans
Source: PLoS One. 2024 Sep 12;19(9):e0302673. doi: 10.1371/journal.pone.0302673 (PMC11392421; doi:10.1371/journal.pone.0302673)

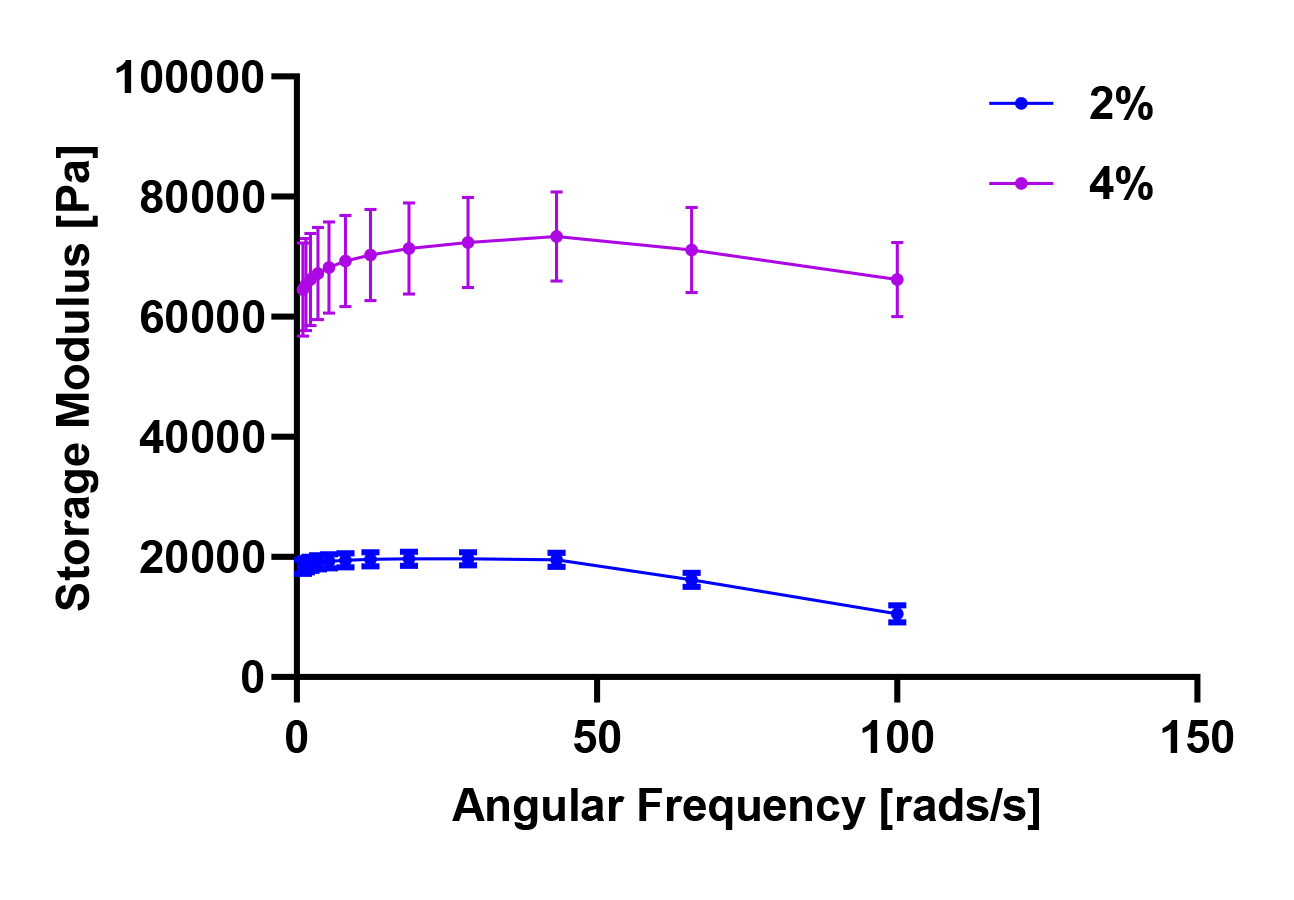

Supplement: S1 Fig — 2% and 4% agar-based solid NGM plates were made as described in Materials and Methods. Stiffness of each agar plate was measured using an oscillatory rheometer. Dots represents mean and lines represent standard deviation. (TIF) [file pone.0302673.s001.tif]

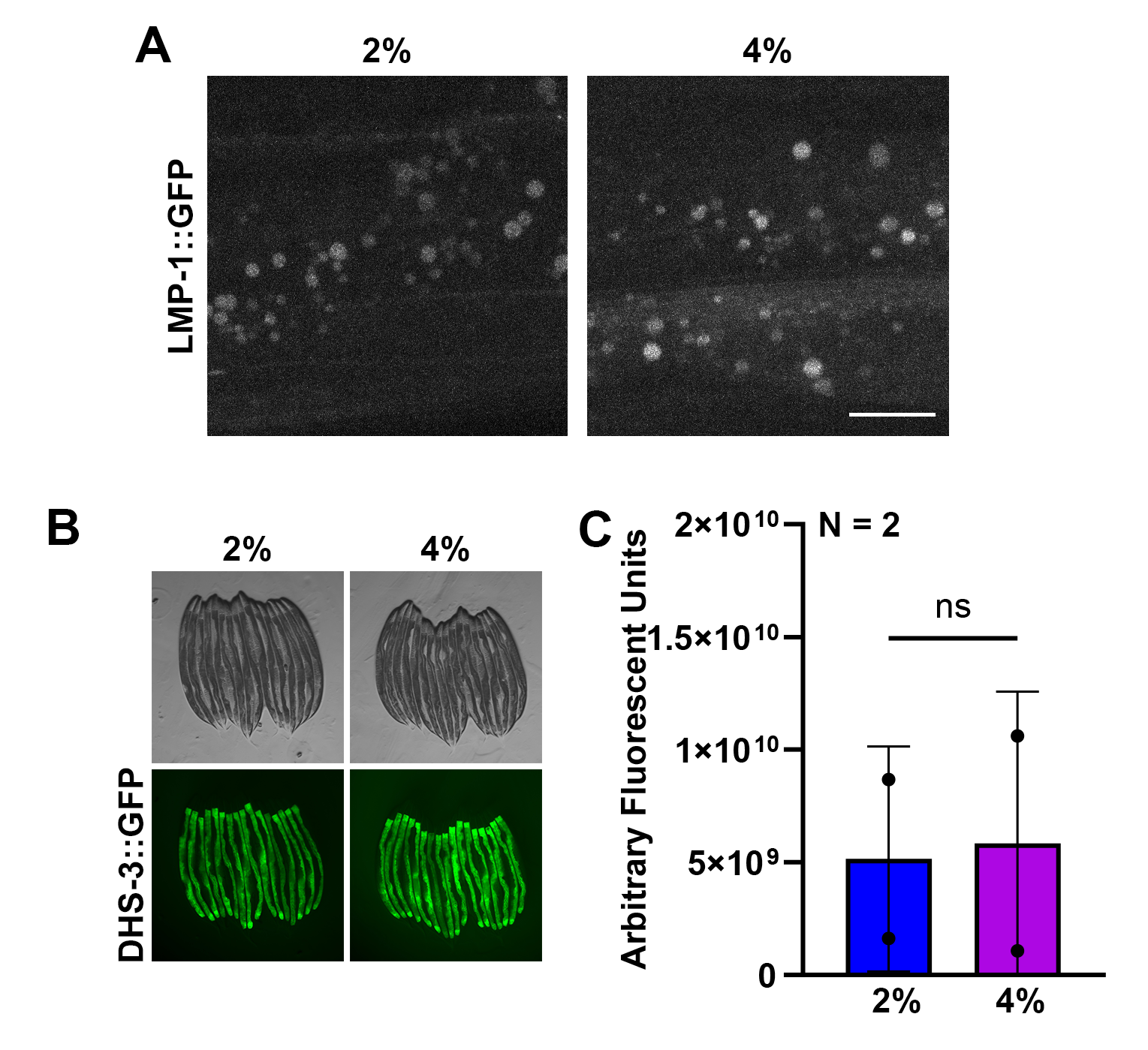

Supplement: S2 Fig — (A) Representative max projection fluorescent images of lysosomes by visualization of LMP-1::GFP. Images were captured on a Leica Stellaris system using optimized z-slices. (B) Representative fluorescent images of lipid droplets by visualization of DHS-3::GFP. For A-B, animals were grown on empty vector (EV) RNAi bacteria from L1 and imaged at day 1 of adulthood. Scale bar is 10 μm. (C) Quantification of B. Arbitrary fluorescent units are integrated fluorescent densities as measured by imageJ Fiji. Data is representative of 2 independent replicates. (TIF) [file pone.0302673.s002.tif]

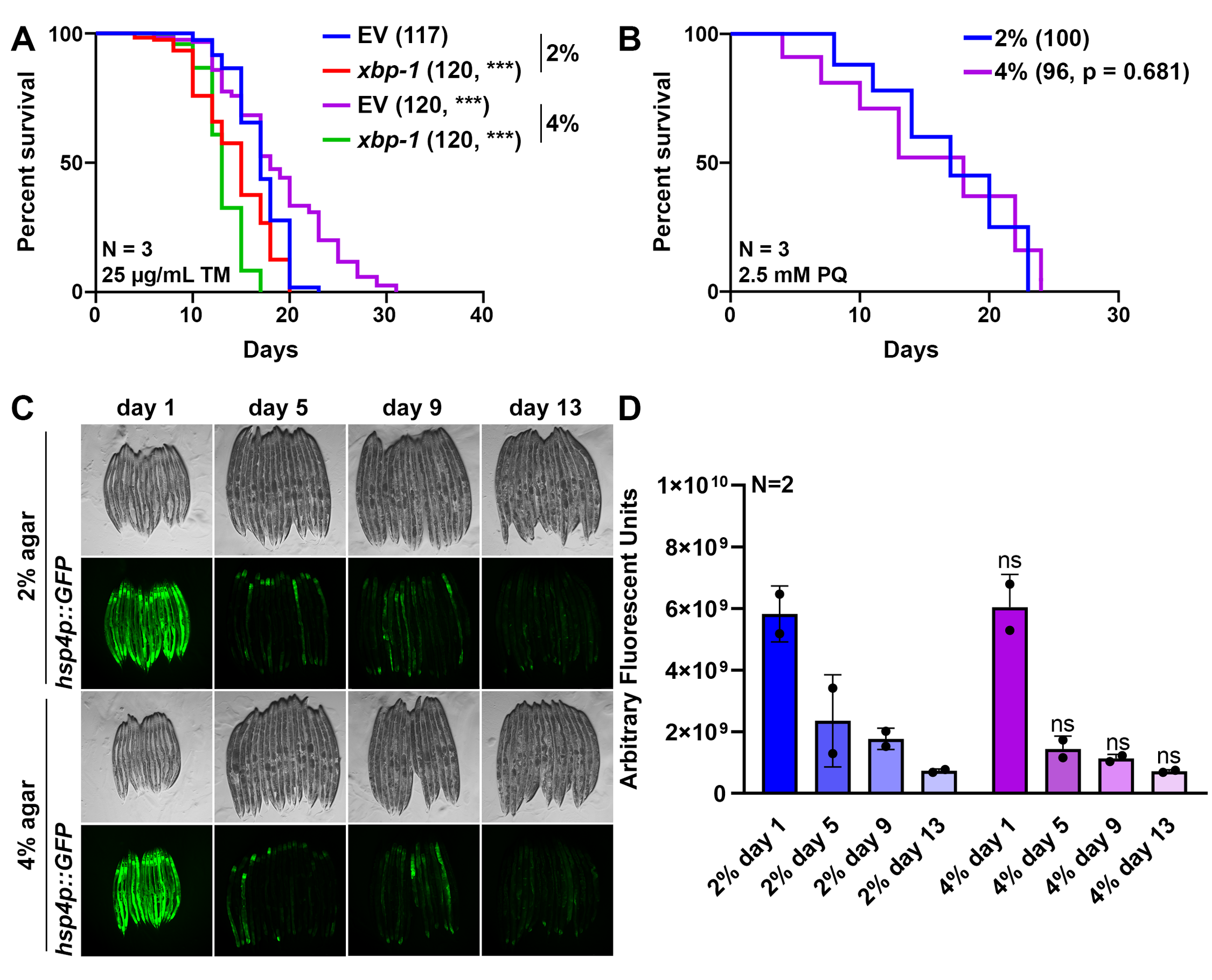

Supplement: S3 Fig — (A) N2 wild-type animals grown on empty vector (EV) or xbp-1 RNAi bacteria on either control (2%) or stiff (4%) agar plates from L1. Animals were transferred onto the same RNAi and agar concentration plates containing 25 μg/mL tunicamycin (TM). Lifespans were scored every 2 days. Data is representative of 3 biological replicates, sample size is represented in the legend in parentheses, and statistical analysis is available in S1 Table. *** p < 0.0001 (B) N2 wild-type animals grown on EV RNAi bacteria on either control (2%) or stiff (4%) agar plates from L1. Animals were moved to plates containing 2.5 mM paraquat (PQ) on day 1 of adulthood and survival was scored every 2 days. Data is representative of 3 biological replicates, sample size is represented in the legend in parentheses, and statistical analysis is available in S2 Table. n.s. = not significant, p = 0.681. (C) Representative fluorescent images of day 1, 5, and 9 adult animals expressing hsp-4p::GFP grown on EV RNAi bacteria from L1. Animals were transferred onto plates containing 25 μg/mL tunicamycin 24 hours prior to imaging on L4, day 4, and day 8. (D) Quantification of (C) where arbitrary fluorescent units are integrated fluorescent intensity measurements using ImageJ Fiji, dots are independent replicates, and lines represent standard deviation. Data is representative of 2 independent replicates. ns = not significant, p > 0.05 comparing age-matched animals grown on 2% or 4% agar. (TIF) [file pone.0302673.s003.tif]

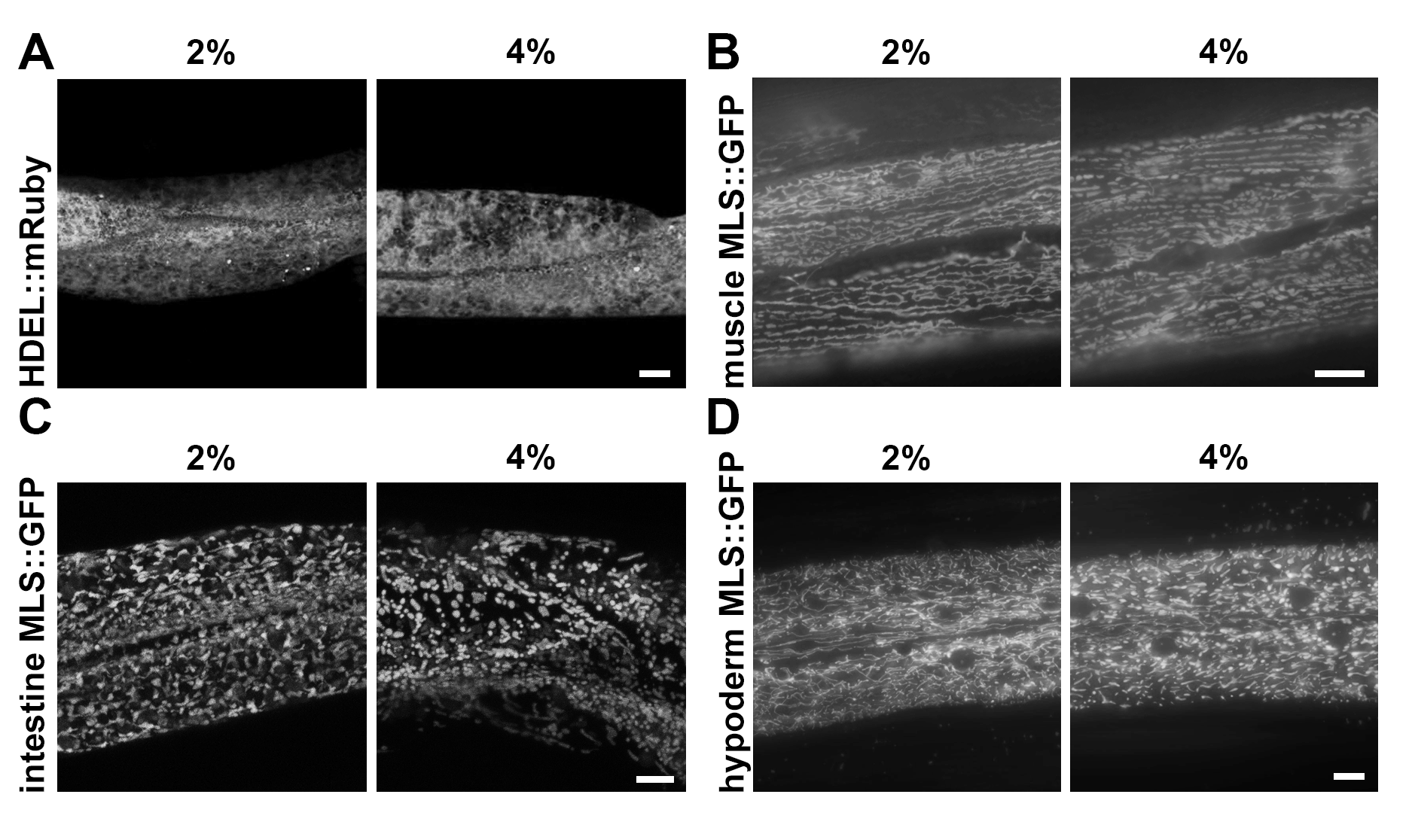

Supplement: S4 Fig — (A) Representative max projection fluorescent images of the endoplasmic reticulum in the intestine by visualization of HDEL::mRuby. Images were captured on a Leica Stellaris system using optimized z-slices. Animals were grown on empty vector (EV) RNAi bacteria from L1. Animals were moved onto 25 μg/mL tunicamycin containing plates at L4 and imaged at day 1 of adulthood. (B) Representative fluorescent images of body wall muscle mitochondria (myo-3p::MLS::GFP) are shown. Animals were grown on EV RNAi bacteria from L1. Single-slice images were captured on a Leica THUNDER Imager. Scale bar is 10 μm. (C) Representative max-projection fluorescent images of intestinal mitochondria (gly-19p::MLS::GFP) are shown. Animals were grown on EV RNAi bacteria from L1. Z-stack images were captured on a Leica THUNDER Imager using system-optimized z-slices. Scale bar is 10 μm. (D) Representative max-projection fluorescent images of hypodermal mitochondria (col-19p::MLS::GFP) are shown. Animals were grown on EV RNAi bacteria from L1. Z-stack images were captured on a Leica Stellaris using system-optimized z-slices. Scale bar is 10 μm. (TIF) [file pone.0302673.s004.tif]

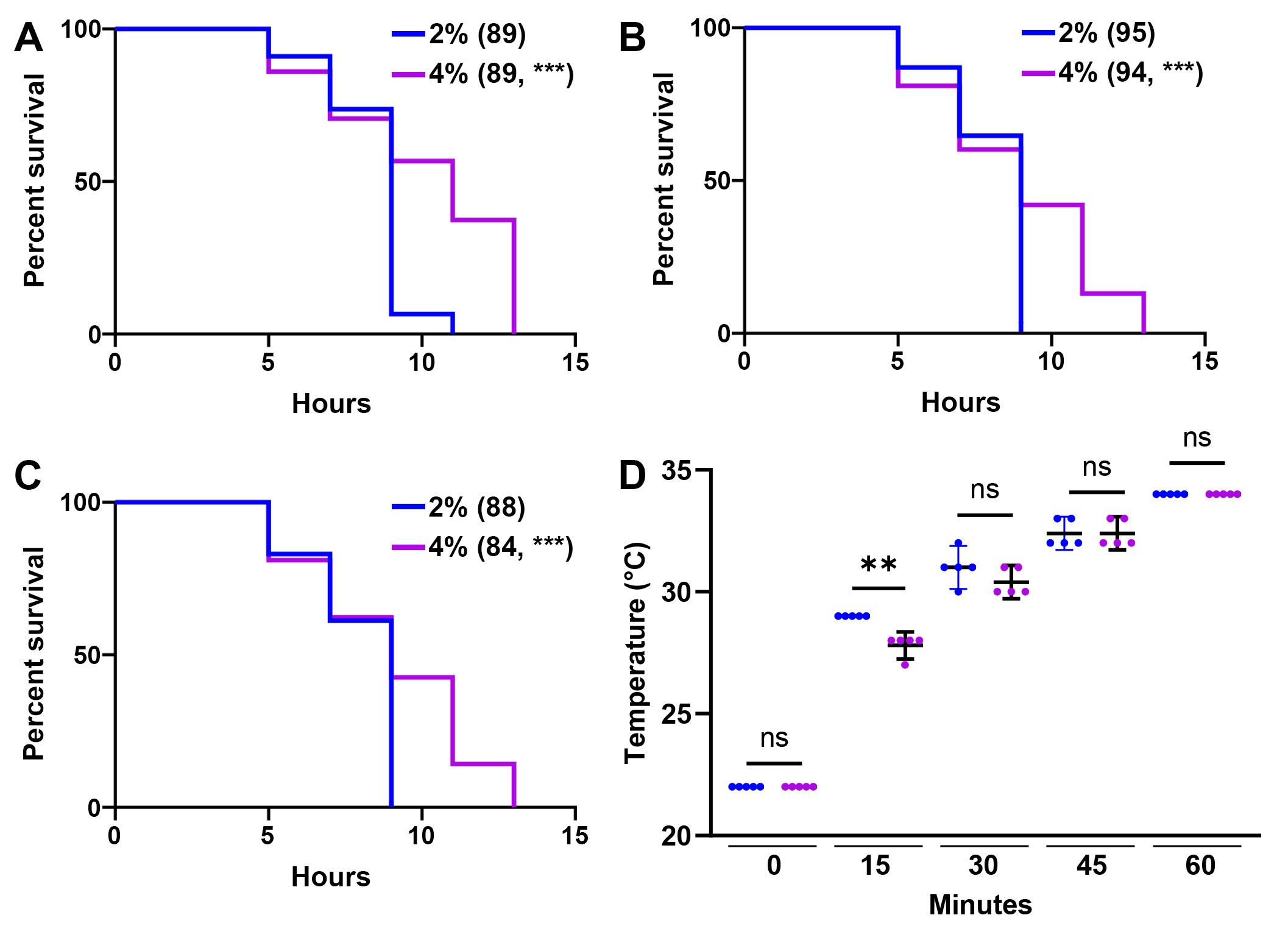

Supplement: S5 Fig — (A-C) N2 wild-type animals grown on empty vector (EV) RNAi bacteria on either control (2%) or stiff (4%) agar plates from L1. Animals were moved to 34°C on day 1 of adulthood and survival was scored every 2 hours. All three biological replicates are shown to highlight differences in maximal thermotolerance, despite similarities in median thermotolerance in some replicates. Sample size is represented in the legend in parentheses and statistical analysis is available in S2 Table. *** p < 0.0001 (D) 2% and 4% plates were placed in a 34°C incubator and the temperature of the plates was measured using an infrared thermometer every 15 minutes. Each dot represents one plate and lines represent mean and standard deviation. ns = not significant, p > 0.05; ** = p < 0.01 using Mann Whitney testing. (TIF) [file pone.0302673.s005.tif]
