# Supplementary material for: Growth on stiffer substrates impacts animal health and longevity in C. elegans
Source: PLoS One. 2024 Sep 12;19(9):e0302673. doi: 10.1371/journal.pone.0302673 (PMC11392421; doi:10.1371/journal.pone.0302673)
Supplement: S3 Table — (DOCX) [file pone.0302673.s008.docx]

| **Strains used in this study** | | |
| --- | --- | --- |
| *C. elegans:* Bristol (N2) strain as wild type (WT) | CGC | N2 |
| *C. elegans:* CL2070: *dvIs70[pCL25 (hsp-16.2p::GFP), pRF4(rol-6)]* | CGC |  |
| *C. elegans*: CL2166: *dvIs19[pAG15(gst-4p::GFP::NLS)] III* | CGC |  |
| *C. elegans*: AGD1664: *uthSi17[myo-3p::MLS::GFP::unc-54 3’UTR::cb-unc-119(+)] I; unc-119(ed3) III* | [1] |  |
| *C. elegans*: AGD1988: *zcIs13[hsp-6p::GFP]* | [2] | SJ4100 6x backcross |
| *C elegans*: AGD2053: *zcIs4[hsp-4P::GFP]V* | [3] |  |
| *C. elegans:* AGD2192: *unc-119(ed3) III; uthSi60[vha-6p::ERss::mRuby::HDEL::unc-54 3'UTR cb-unc-119(+)] IV;: AGD2192:* | [4] |  |
| *C. elegans*: DA2123: *adIs2122[lgg-1p::GFP::lgg-1 + rol-6(su1006)]* | CGC |  |
| *C. elegans*: RT258: *pwIs50[lmp-1::GFP + Cbr-unc-119(+)]* | CGC |  |
| *C. elegans:* RHS10: *ldrIs[dhs-3p::dhs-3::GFP + unc-76(+)]* | This study | LIU1 6x backcross |
| *C. elegans:* RHS41: *uthSi7[myo-3p::LifeAct::mRuby::unc-54 3'UTR::cb-unc-119(+)] IV* | [5] | AGD1651 4x backcross |
| *C. elegans:* RHS42: *uthSi10[col-19p::LifeAct::mRuby::unc-54 3'UTR::cb-unc-119(+)] IV* | [5] | AGD1654 4x backcross |
| *C. elegans:* RHS43: *uthSi13[gly-19p::LifeAct::mRuby::unc-54 3'UTR::cb-unc-119(+)] IV* | [5] | AGD1657 4x backcross |

**References**

1. Daniele JR, Esping DJ, Garcia G, Parsons LS, Arriaga EA, Dillin A. “High-Throughput Characterization of Region-Specific Mitochondrial Function and Morphology.” Sci Rep. 2017;7: 6749. doi:10.1038/s41598-017-05152-z

2. Garcia G, Bar-Ziv R, Averbukh M, Dasgupta N, Dutta N, Zhang H, et al. Large-scale genetic screens identify BET-1 as a cytoskeleton regulator promoting actin function and life span. Aging Cell. 2023;22: e13742. doi:10.1111/acel.13742

3. Higuchi-Sanabria R, Durieux J, Kelet N, Homentcovschi S, de Los Rios Rogers M, Monshietehadi S, et al. Divergent Nodes of Non-autonomous UPRER Signaling through Serotonergic and Dopaminergic Neurons. Cell Rep. 2020;33: 108489. doi:10.1016/j.celrep.2020.108489

4. Daniele JR, Higuchi-Sanabria R, Durieux J, Monshietehadi S, Ramachandran V, Tronnes SU, et al. UPRER promotes lipophagy independent of chaperones to extend life span. Science Advances. 2020;6: eaaz1441. doi:10.1126/sciadv.aaz1441

5. Higuchi-Sanabria R, Paul Rd JW, Durieux J, Benitez C, Frankino PA, Tronnes SU, et al. Spatial regulation of the actin cytoskeleton by HSF-1 during aging. Mol Biol Cell. 2018;29: 2522–2527. doi:10.1091/mbc.E18-06-0362
