# Supplementary material for: Growth on stiffer substrates impacts animal health and longevity in C. elegans
Source: PLoS One. 2024 Sep 12;19(9):e0302673. doi: 10.1371/journal.pone.0302673 (PMC11392421; doi:10.1371/journal.pone.0302673)
Supplement: Attachment — Submitted filename: Oorloff-Hruby Response to Reviewers.pdf [file pone.0302673.s009.pdf]

We thank the reviewers and editors for the thorough evaluation of the manuscript and the many helpful suggestions. We have made considerable effort to address all the concerns, most of which were editorial, but have also performed additional experiments to help ameliorate concerns. A detailed point by point is provided below – overall, changes focused on toning down conclusions to better reflect the data presented and to better incorporate literature from the field as requested by reviewers. Importantly, we have experimentally confirmed that 4% agar has a significantly stiffer substrate and also have made all conclusions of mechanical stress's impact on physiology purely speculative in this study. All changes are highlighted in red in the "tracked changes" version of the revised document.

Reviewer #1: This paper considers the hypothesis that growing animals on stiffer substrates will have an impact on their physiology by mechanotransduction pathways. The data and experiments are appropriately presented and the paper is clearly written, however, I think the way the experiments are framed isn't supported by the data. Specifically, changing the agar concentration from 2-4% will increase stiffness, but it may also change other aspects of the experiments that could have an impact on worm behaviour and physiology. For example, the plates may dry differently and worms are known to be sensitive to humidity. Different concentrations of agar could affect bacterial growth or physiology that in turn affects the worms. Indeed, even a subtle change on bacterial physiology could explain the small changes in worm phenotypes. Ruling this out may be difficult, but without much better controls, I see how the authors can conclude that the observed effects are mechanical.
This is a fantastic point brought up by both reviewers, and thus we have presented limitations of our study using 2% and 4% agar throughout both the introduction and discussion sections. These are detailed better throughout the point by point below. Importantly, we have rewritten the manuscript to focus primarily on 2% and 4% conditions and discuss the data as comparison of agar % or an increase in stiffness for 4% plates as we have confirmed experimentally and only speculate on the impact of mechanical stress.

While we agree that ruling out all differences (water content, humidity, etc.) would be challenging, we have performed a lifespan experiment on dead bacteria on 2% and 4% and show that even when using dead bacteria, growth on 4% displays a mild increase in lifespan similar to trends when using live bacteria, suggesting that at least changes in bacterial growth or physiology are likely not the driving factor.

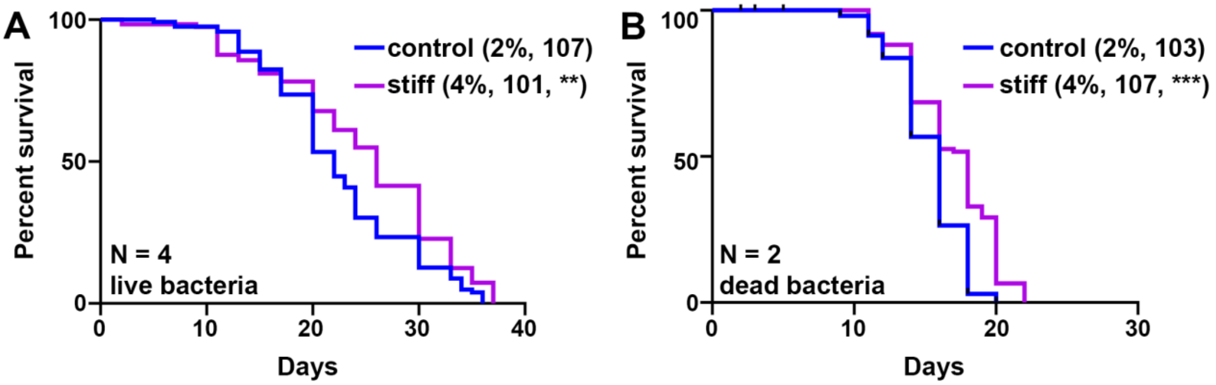

Related to this, I don't share the authors shock at seeing only subtle effects of stiffness on worm physiology. Unlike adherent cells which bind to their substrates through integrins and pull on them, worms sit on top of agar in both the 2 and 4% conditions. Similarly, I wouldn't be shocked to see that people with thick carpets have very different physiology to people with thin carpets.

And if they did, I wouldn't look for changes in mechanotrasduction related genes to explain any difference. What is the model for an impact on the worms? My expectation is that the forces the cells in the worm experience are dominated by muscle contraction not direct interaction with the substrate. If anything, because worms sink a little bit into the agar, I might imagine more mechanical stress in the softer condition (a bit like walking in deeper mud).
We appreciate the reviewer's comments and have removed all instances of shock, surprise, etc. However, we would like to argue that growth of *C. elegans* on 2% or 4% is not the same as a person walking on a thicker carpet. *C. elegans* do not walk, and thus their entire bodies directly interact with the environment, including exchange of solutes and water with the culture medium surface. Therefore, there is no good analogy of this type of lifestyle with humans, but the best attempt at it would be something similar to what the reviewer mentioned as being in mud. However, it wouldn't be walking, but rather a person crawling within the mud with the entire body in contact with either the softer or stiffer mud. This would likely impact the mechanotransduction of the epithelial cells in contact with the mud, as mechanosensory neurons, touch neurons, and even epithelial cells to a degree do respond to the external mechanical stimulus.

Although a good analogy to humans is very hard to make in terms of growth on 2% or 4% agar, another lab has shown that differences in culture media firmness (i.e., agar %) can have physiological impact on *C. elegans* in a relevant disease model to humans. They argue that animals on stiffer substrates experience mechanical stress due to their crawling action on a stiffer substrate inducing more stress on them. Importantly, in muscular dystrophy models of *C. elegans*, growth on 5% agar resulted in a significant increase in muscle degeneration in these models (https://pubmed.ncbi.nlm.nih.gov/26358775/). Although the many limitations brought up by this reviewer are still valid, this study does at least show a potential link of this method to human health and disease. We have now included this report in our revised manuscript.

Given the difficulty of isolating stiffness from other properties of the system, the results and discussion should be framed around agar concentration differences rather than stiffness differences. Of course it's fine to speculate that the differences are due to stiffness, but without many more controls and a model of how stiffness could plausibly stress worms mechanically, it should be clearly marked as speculation.
As suggested by the reviewer, we have rewritten all conclusions of the manuscript to detailing specifically agar % differences and stiffness and only speculate on how some of the changes we observe could potentially be due to mechanical stress. Most importantly, we have measured the stiffness of 2% and 4% plates and show that 4% plates are 4x fold stiffer than 2%, thus providing more direct evidence that the increase in agar percentage does reflect increased stiffness. However, as the reviewer correctly stated, there are still many caveats to the method, and thus we have ensured that our discussions were toned down significantly.

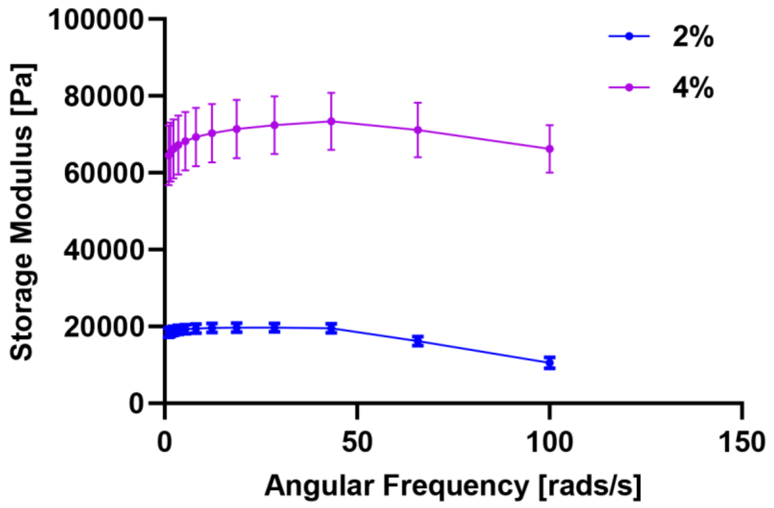

Personally I would also remove the statements around being 'surprised' or 'shocked' that worms were 'wildly unaffected', but it's just a question of style.
All instances of surprised or shocked or other related text has been removed.

Minor points:

-Does tunicamycin interact with agar? Is it possible some of it is sequestered by the higher agar concentration or that it's diffusion is slowed so that there is a slight depletion at the agar surface where the worms are?
This is a fantastic point, although it is extremely challenging to test directly. However, we have made an effort to at least address this indirectly. We used a previously validated reporter for the UPR^{ER}, *hsp-4p::GFP,* which was also used in this study. Under conditions of ER stress, this reporter is induced, and as previously shown, this reporter can be activated using tunicamycin. This activation also declines during aging (https://pubmed.ncbi.nlm.nih.gov/23791175/). Here, we show that the activation of this reporter is not different in 2% vs. 4% at any age, which suggests that the tunicamycin-agar interaction is likely not impacting our results, and that the difference in tunicamycin resilience is likely due to the growth on the higher agar percentage.

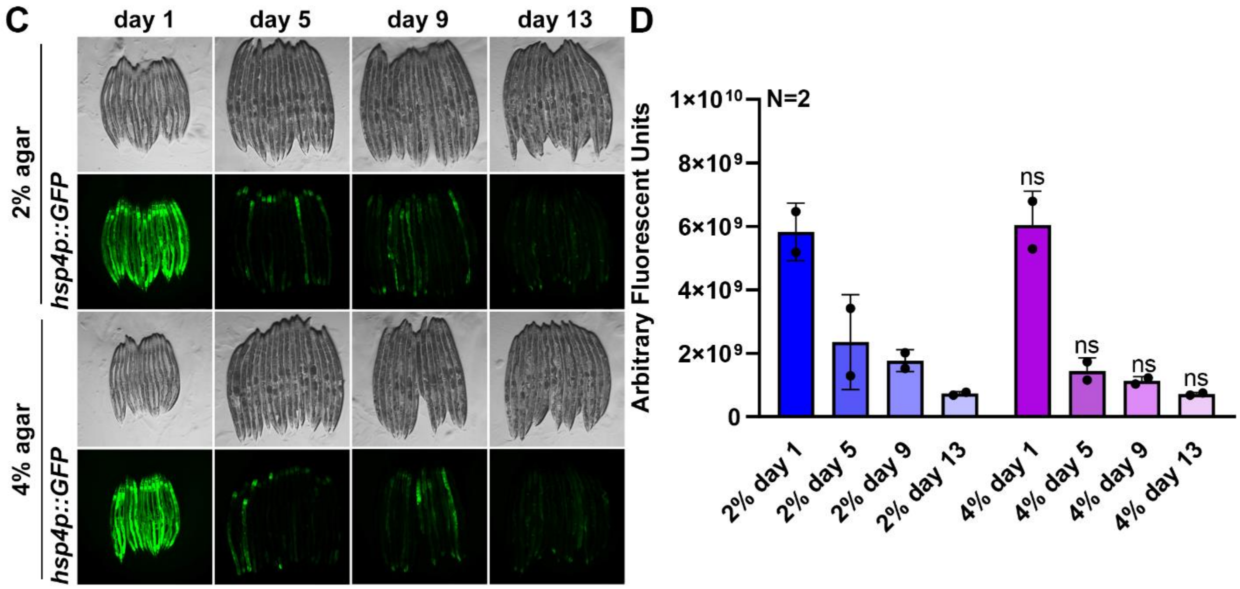

-For Fig 3B wouldn't a plot of values be more useful than a heatmap? Also maybe easier to read if the genes are sorted by expression change?

We have added all raw values to the heatmap so that all the data is as transparent as possible.

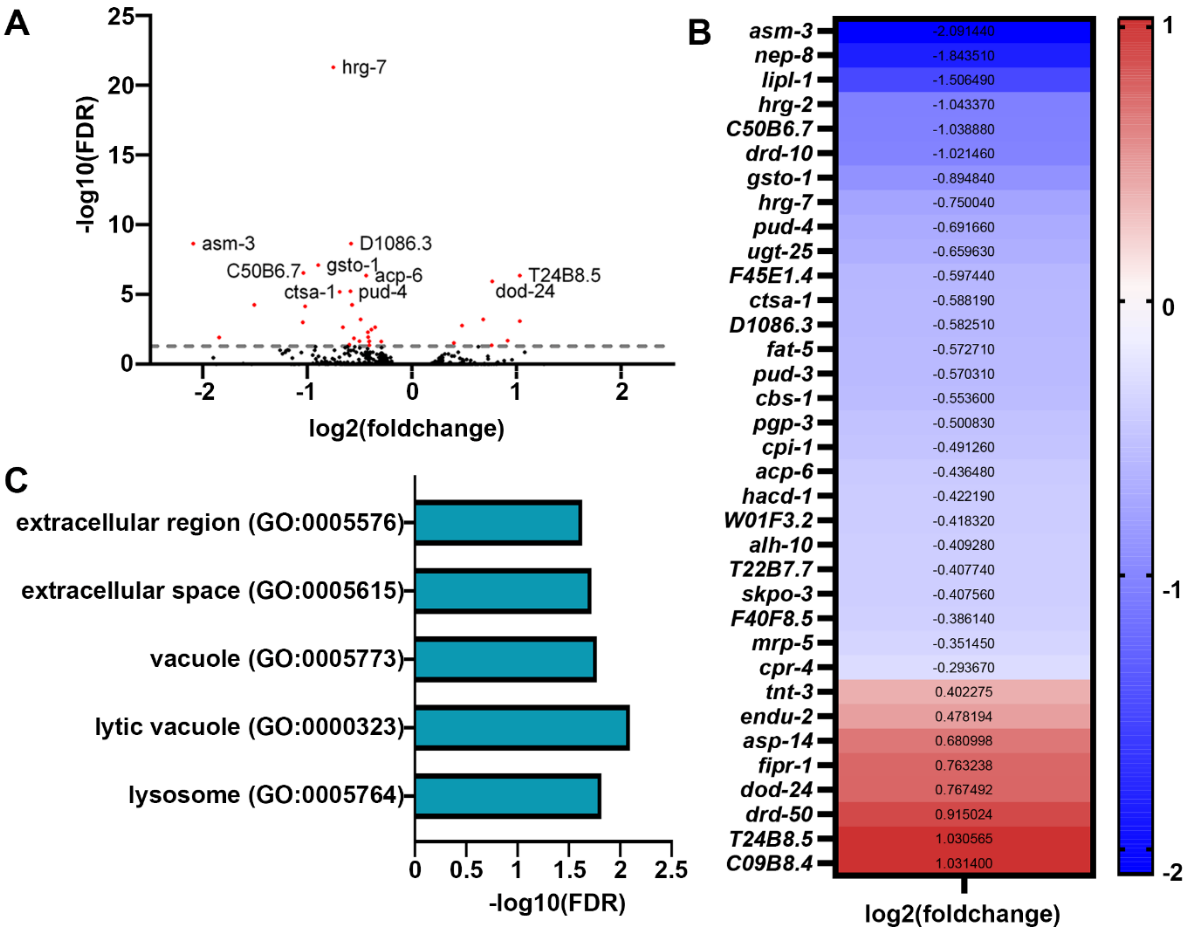

-The authors state that “stiffer substrates can result in integrin-dependent remodeling of the mitochondria and actin cytoskeleton in both human cells [13] and C. elegans [31]", however these results did not show that stiffer substrates affect worm mitochondria, they show that integrin signalling in worms can affect mitochondria. The discussion of these results is fine elsewhere in the paper.

The sentence has been rewritten to make it clearer that previous work was solely on integrin signaling and our current work is on differences in agar percentage.

-The statement that "actin stability [...] is likely responsible for the mild increase in longevity" isn't sufficiently supported by the data. Many other things could be going on including mild calorie restriction because of differences in bacterial growth.

We have toned down this section to describe changes in actin stability as only one possible mechanisms for the lifespan extension.

-From the discussion: "Perhaps animals grown on stiffer substrates may not activate integrin signaling to a sufficient level to induce transcriptional changes but do increase mechanosignaling pathways enough to promote actin quality". A direct connection here seems unlikely to me without some model for how stiffer substrates would activate a mechanosignaling pathway except perhaps in a sensory neuron.

While we understand the reviewers concern, there are others that have shown that growth on different agar percentages can directly impact *C. elegans* physiology (https://pubmed.ncbi.nlm.nih.gov/26358775/). What this paper argues is that crawling on a stiffer substrate induces mechanical stress onto the animal's body. However, the limitation on impact of the substrate stiffness on actin quality is still relevant, so we have removed this sentence from the manuscript and make involvement of actin purely speculative.

-From the discussion: "It is imperative that NGM agar plates not be stored long-term, as desiccation of plates can indirectly increase agar precentage relative to water content. These plates will be stiffer and can change your biological data, especially for phenotypes similar to those we assayed in this study. Another very important consideration is to standardize an agar choice in the lab since multiple different agar sources have dramatically different stiffnesses". I agree with the advice, but I doubt stiffness is the main contributor and I don't think it's supported by the data presented here.
First, we have confirmed that 4% plates are significantly stiffer than 2% plates. We have also toned down this section to state that stiffness of the plate is just one of many factors that can impact experiments.

Reviewer #2: Mechanical signaling plays a pivotal role in many biological phenomena, including cell growth, differentiation, and aging. In their study, Oorloff et al. employ the C. elegans model to investigate the impact of external mechanical stress (2% vs. 4% agar gel) on aging, stress responses, and cytoskeletal proteins. The authors report that external mechanical stress modestly extends lifespan at the expense of reduced locomotor activity and reproductive capacity. Although several stress responses, as indicated by transcriptional markers, appeared unaffected, RNA sequencing revealed minimal gene expression changes. The authors suggest that actin dynamics may partially mediate the observed effects of mechanical stress on aging. However, due to the marginal nature of these effects, they conclude that mechanical stress has a limited impact on the studied phenotypes.

While this paper might capture the interest of the C. elegans research community, the core assertion that environmental mechanical stress significantly influences these outcomes lacks robust support, with deficiencies in biological context explanation, control usage, and data quantification. Several critical issues require attention:

We thank the reviewer for the many helpful suggestions and have toned down the conclusions of our manuscript to address the reviewer's concerns as detailed below.

Major concerns:

1. While using C. elegans to study the impact of mechanical stress on animal health and behavior is valid, the authors oversimplify the mechanistic implications of environmental and extracellular matrix-derived mechanical stresses. The introduction may lead readers to assume uniform effects of mechanical forces at both organismal and cellular levels. The discussion acknowledges the potential confounding effect of the C. elegans cuticle, but this issue warrants more extensive consideration in both the introduction and discussion sections.
This is a great suggestion and we have included a limitations section in the introduction in addition to the discussion section. Importantly, all mention of mechanical stress has been written as a speculation and now we refer to the differences in agar % and increased stiffness throughout the text.

2. The authors would like to use C. elegans as a model to study the effect of external

mechanical stress on physiology. It is noteworthy that the body stiffness gradually becomes softer in aging C. elegans (PMID:32098962), which contradicts to examples in introduction. However, this piece of evidence suggests that mechanical forces might be an indicator of aging or even likely involve in aging. It is sad that this paper was not included and mentioned in the introduction and discussion. In fact, this work shows that body stiffness drastically changes over 20 folds along aging, suggesting that epithelial cells are highly elastic to maintain body integrity and probably have different sensitivities to external mechanical stress. It also raises an issue that whether 2-fold changes of agar can reveal biological alterations from the current setting.
We thank the reviewer for bringing this paper to our attention and we have included this in the manuscript. We have also performed AFM on 2% vs. 4% agar and show that although there is only a 2-fold change in agar %, this actually results in a 4-fold increase in stiffness. In addition, another *C. elegans* study showed that differences in culture media firmness (i.e., agar %) can have physiological impact on *C. elegans* in a relevant disease model to humans. Specifically, in muscular dystrophy models of *C. elegans*, growth on 5% agar resulted in a significant increase in muscle degeneration in these models (https://pubmed.ncbi.nlm.nih.gov/26358775/).

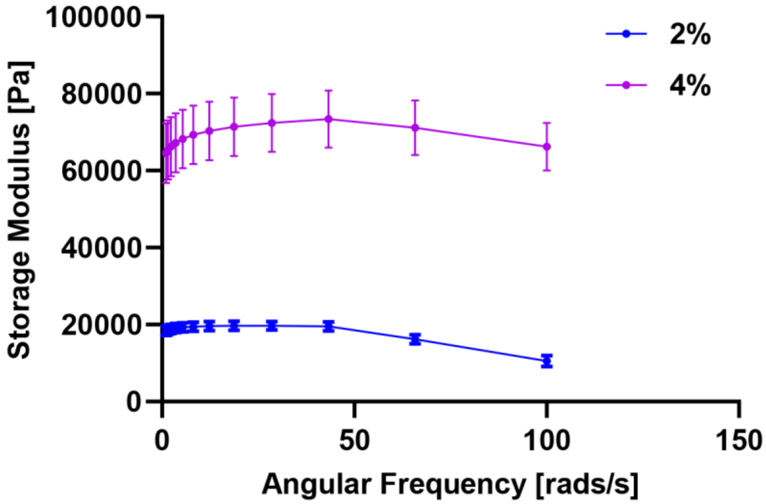

3. Mechanical forces also influence the bacteria that serve as food for C. elegans (PMID: 36712798). The potential indirect effects of mechanical forces via changes in food source quality or abundance necessitate additional controls to distinguish these influences from direct interactions between the agar and C. elegans.
This is a great point. While we believe that assessing all changes in bacterial physiology is out of the scope of the manuscript, we have performed a lifespan experiment on dead bacteria on 2% and 4% and show that even when using dead bacteria, growth on 4% displays a mild increase in lifespan similar to trends when using live bacteria, suggesting that at least changes in bacterial growth or physiology are likely not the driving factor.

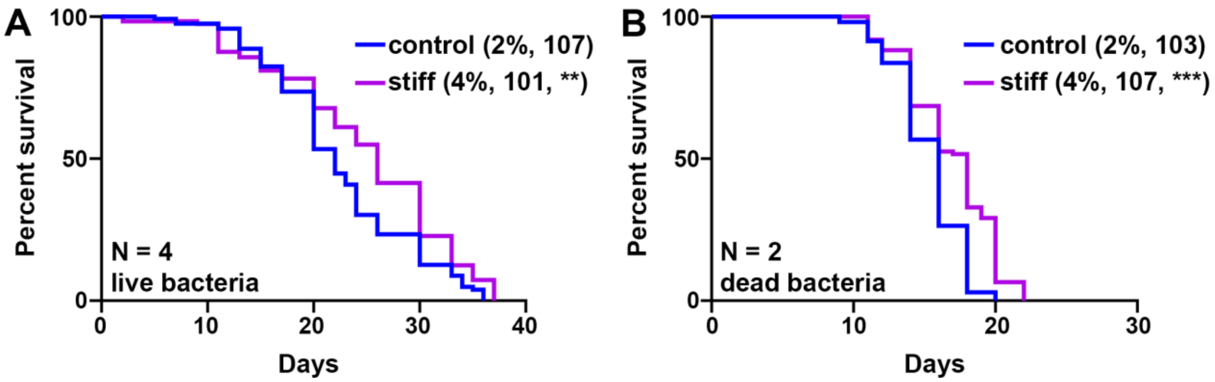

4. Despite claims that stress responses were generally unaffected, observed data from fluorescent images suggest increased stress responses in stiffer substrates under stress conditions. Quantitative data should be provided to substantiate this conclusion.

All fluorescent imaging data has been quantified and added into the manuscript. We have expanded in this section that although some observed differences are visible in the fluorescent images, none of them were statistically significant when quantified.

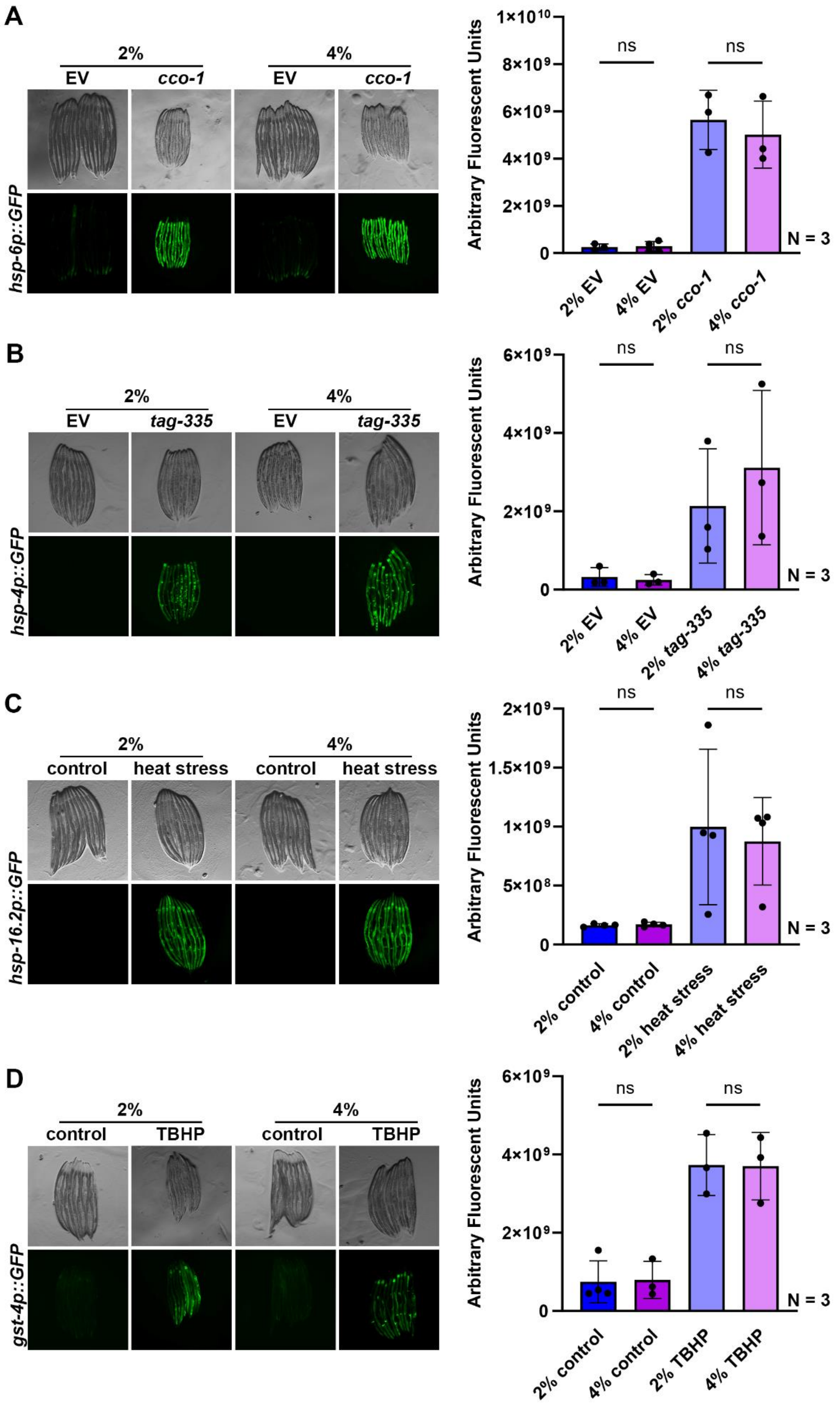
A
2%
EV
cco-1
4%
EV
cco-1
hsp-6p::GFP
Arbitrary Fluorescent Units
1×10¹⁰
8×10⁹
6×10⁹
4×10⁹
2×10⁹
0
ns
ns
N = 3
2% EV
4% EV
2% cco-1
4% cco-1
B
2%
EV
tag-335
4%
EV
tag-335
hsp-4p::GFP
Arbitrary Fluorescent Units
6×10⁹
4×10⁹
2×10⁹
0
ns
ns
N = 3
2% EV
4% EV
2% tag-335
4% tag-335
C
2%
control
heat stress
4%
control
heat stress
hsp-16.2p::GFP
Arbitrary Fluorescent Units
2×10⁹
1.5×10⁹
1×10⁹
5×10⁸
0
ns
ns
N = 3
2% control
4% control
2% heat stress
4% heat stress
D
2%
control
TBHP
4%
control
TBHP
gst-4p::GFP
Arbitrary Fluorescent Units
6×10⁹
4×10⁹
2×10⁹
0
ns
ns
N = 3
2% control
4% control
2% TBHP
4% TBHP

5. The claim that actin stability is influenced by agar stiffness is based solely on the abundance of actin, which does not directly reflect actin dynamics. Evidence supporting this claim or a revision of the statement to more accurately reflect the findings is needed.
We apologize for the confusion and have changed the term “actin stability” to “actin organization”. We are only imaging the organization of the actin cytoskeleton and actually do not have any measurements of actin abundance (because LifeAct is an artificial actin-binding protein, the levels of LifeAct are not directly related to actin abundance). In our original manuscript, we were careful to not have any mention of actin dynamics and have attempted to clarify this even further in the revised manuscript.

Minor concerns:
1. The number of experiments conducted (N) and statistical analyses are missing in most figures. Consider relocating this information from figure legends directly into the figures for better clarity.
We thank the reviewer for this comment and have added sample size and number of biological replicates wherever possible within the manuscript for all quantified data.

2. Allele names should be italicized to adhere to standard scientific formatting (in methods).
We apologize for this error and have corrected the allele names.

3. A dedicated statistical methods section is absent in the methodology description, which is crucial for reproducibility and validation of the results.
We have added a dedicated statistical methods section and have also ensured that all statistics are presented in every figure legend.
